# SeaFlow data v1, high-resolution abundance, size and biomass of small phytoplankton in the North Pacific

**DOI:** 10.1038/s41597-019-0292-2

**Published:** 2019-11-22

**Authors:** François Ribalet, Chris Berthiaume, Annette Hynes, Jarred Swalwell, Michael Carlson, Sophie Clayton, Gwenn Hennon, Camille Poirier, Eric Shimabukuro, Angelicque White, E. Virginia Armbrust

**Affiliations:** 1 School of Oceanography, University of Washington, Seattle, WA 98195 USA; 2 Faculty of Biology, Technion – Israel Institute of Technology, Haifa, 3200003 Israel; 3 Department of Ocean, Earth and Atmospheric Sciences, Old Dominion University, Norfolk, VA 23529 USA; 4 College of Fisheries and Ocean Sciences, University of Alaska Fairbanks, Fairbanks, AK 99775 USA; 5 Monterey Bay Research Institute, 7700 Sandholdt Rd, Moss Landing, CA USA; 6 Ocean EcoSystems Biology Unit, GEOMAR Helmholtz Centre for Ocean Research Kiel, Dusternbrookerweg 20, Kiel, Germany; 7 Department of Oceanography, Daniel K. Inouye Center for Microbial Oceanography: Research and Education (C-MORE), University of Hawai’i at Manoa, Honolulu, HI 96822 USA

**Keywords:** Microbial ecology, Marine biology

## Abstract

SeaFlow is an underway flow cytometer that provides continuous shipboard observations of the abundance and optical properties of small phytoplankton (<5 *μ*m in equivalent spherical diameter, ESD). Here we present data sets consisting of SeaFlow-based cell abundance, forward light scatter, and pigment fluorescence of individual cells, as well as derived estimates of ESD and cellular carbon content of picophytoplankton, which includes the cyanobacteria *Prochlorococcus*, *Synechococcus* and small-sized *Crocosphaera* (<5 *μ*m ESD), and picophytoplankton and nanophytoplankton (2–5 *μ*m ESD). Data were collected in surface waters (≈5 m depth) from 27 oceanographic cruises carried out in the Northeast Pacific Ocean between 2010 and 2018. Thirteen cruises provide high spatial resolution (≈1 km) measurements across 32,500 km of the Northeast Pacific Ocean and 14 near-monthly cruises beginning in 2015 provide seasonal distributions at the long-term sampling site (Station ALOHA) of the Hawaii Ocean Time-Series. These data sets expand our knowledge of the current spatial and temporal distributions of picophytoplankton in the surface ocean.

## Abstract (summary)

**Table:** 

Measurement(s)	planktonic material • temperature of water • salinity • light irradiance • plankton size distribution
Technology Type(s)	flow cytometer • Temperature Sensor Device • salinometer • photometer
Factor Type(s)	time • location
Sample Characteristic - Organism	Cyanobacteria • Micromonas pusilla • Navicula transitans • Thalassiosira pseudonana • Thalassiosira weissflogii • Geminifera cryophila • Crocosphaera • Synechococcus sp. WH 8012 • Synechococcus sp. WH 6501 • Prochlorococcus eMED4 • Prochlorococcus eMIT9312 • Prochlorococcus AS9601 • Prochlorococcus 1314
Sample Characteristic - Environment	oceanic sea surface microlayer biome • sea water
Sample Characteristic - Location	North Pacific Ocean

Machine-accessible metadata file describing the reported data: 10.6084/m9.figshare.10110755

## Background & Summary

Marine phytoplankton are responsible for about half of the planet’s annual production of oxygen and organic carbon, and thus play a significant role in mediating global biogeochemical cycles^1^. Quantitative information on the temporal and spatial distributions of phytoplankton populations in the ocean is critical for understanding how these organisms interact with their environments. Individual phytoplankton species range in diameter from ≈0.6 *μ*m to over a millimeter^2^, with a predominance of the smaller phytoplankton (less than a few micrometers in size) in open ocean environments. In oligotrophic subtropical gyres, phytoplankton communities are numerically dominated by the cyanobacteria of the genus *Prochlorococcus* (<1 *μ*m in diameter), which are well-adapted to low nutrient conditions^3^. The nitrogen gas-fixing cyanobacteria *Crocosphaera* (2–5 *μ*m in diameter) are also sporadically observed in nitrogen-limited subtropical gyres; a portion of the nitrogen fixed by these organisms is made available to other phytoplankton^4^. In colder, more productive subpolar gyres, the cyanobacteria *Synechococcus* (1–2 *μ*m in diameter) and picophytoplankton and nanophytoplankton (2–5 *μ*m in diameter) numerically dominate phytoplankton communities^5,6^.

The abundance and distribution of different groups of phytoplankton reflect a combination of prevailing environmental conditions and resulting food-web dynamics. Flow cytometry is well-suited to mapping the distribution of the small phytoplankton (<5 *μ*m in diameter) because of their relatively high abundance and the innate fluorescence of their pigments; for example, all phytoplankton possess chlorophyll *a* and a subset additionally possess phycoerythrin (e.g., *Synechococcus* and *Crocosphaera*). Models based on compilations of flow cytometry measurements from 1987–2011 predict that the distributions of cyanobacteria, picophytoplankton and nanophytoplankton may change significantly in future oceans^5,6^ as the surface waters warm and nutrient supply is reduced^3^. However, because the dim cellular chlorophyll fluorescence of *Prochlorococcus* in oligotrophic surface waters is near the detection limit of most commercially-available flow cytometers^7^, information on the broad-scale distribution of *Prochlorococcus* in surface waters remains limited.

SeaFlow is a custom-built shipboard flow cytometer developed for high-resolution observations of picophytoplankton in surface waters, including *Prochlorococcus*^8^. SeaFlow eliminates the traditional need for a sheath fluid by employing a unique optical system that relies on three photodetectors, including two position-sensitive detectors, to create a virtual core in the sample stream within which the properties of particles are accurately measured. This enables the instrument to continuously sample surface seawater from a ship’s flow-through seawater system.

**Fig. 1:**
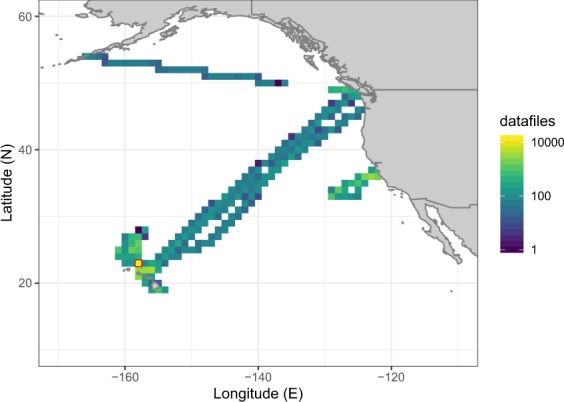
Distribution of the number of data files. Location and number of data files aggregated into 1 degree bins of latitude and longitude. Red outlined square indicates the location of Station ALOHA.

Here, we present SeaFlow datasets consisting of over 69,000 data files collected in surface waters in the Northeast Pacific Ocean (Fig. 1 and Table 1). From 2010–2018, SeaFlow was deployed on 27 cruises conducted across 32,500 km. Data files are aggregated over three-minute intervals to yield a spatial resolution of ≈1 km along the cruise track (for a ship cruising at 11 knots). Beginning in 2015, SeaFlow was deployed on near-monthly cruises in the North Pacific Subtropical Gyre, at or near the long-term Hawaii Ocean Time-series (Station ALOHA, 22.75 degN, 158 degW). Primary data are cell abundances of phytoplankton populations, optical measurements of light scatter, red and orange fluorescence associated with the pigments chlorophyll a and phycoerythrin, respectively. The classification of particles into cell populations was conducted uniformly across all samples using a combination of manual gating and unsupervised clustering algorithms^9^. The data sets were expanded to include equivalent spherical diameter (ESD) and carbon quotas derived from light scatter measurements. ESD was estimated by applying Mie light scattering theory to a combination of flow cytometry calibration beads and cultured organisms of determined size. Carbon quotas were then estimated from ESD using a volume-to-carbon conversion factor^10^. The estimates of cell abundance, light scatter, fluorescence emissions, ESD and carbon quotas include a measurement error based on the uncertainties in the virtual core volume and light scatter conversion. Sample metadata includes location, time, underway sea surface temperature, salinity and photosynthetically active radiation (PAR) and were merged with the SeaFlow data sets. These data are available without restrictions at the Zenodo open access research data repository.

**Table 1:** List of datasets and associated cruise and geolocation metadata.

Cruise	Year	Month	Location	# Datafiles
TN248	2010	May	Gulf of Alaska	1734
TN271	2011	October	Seattle - Hawaii	3596
CN11ID	2011	October	California current	5131
TN280	2012	May	Washington coast	2690
CN12ID	2012	September	California current	3979
TN292	2013	March	Seattle - Hawaii	3134
CN13ID	2013	October	California current	4359
KM1427	2014	December	Aloha	1483
KM1502	2015	March	Portland - Hawaii	3799
KM1508	2015	May	Aloha	1789
KM1510	2015	June	Aloha	1222
KM1512	2015	July	Aloha	1337
KOK1512	2015	September	Aloha	510
KOK1515	2015	October	Aloha	1271
KM1518	2015	November	Aloha	1475
KM1601	2016	January	Aloha	1550
KM1602	2016	February	Aloha	1590
KM1603	2016	March	Aloha	562
KOK1604	2016	April	Aloha	1630
KOK1607	2016	May	Aloha	720
KOK1608	2016	July	Aloha	1645
KOK1609	2016	August	Aloha	1700
KM1708	2017	June	Aloha	1185
KM1709	2017	July	Hawaii	7581
KOK1806	2018	July	Hawaii	1556
FK180310-1	2018	March	Hawaii	5264
FK180310-2	2018	March	Hawaii	6151

## Methods

### Data collection

Each ship’s flow-through seawater system provided continuous flow of seawater collected at an assumed depth of ≈5 m (3–8 m depending on the research vessel and sea state). The water passed through a 100-*μ*m stainless steel-mesh filter before it was sampled to prevent clogging of the 200-*μ*m SeaFlow sampling nozzle.

A real-time broadcast of position, time, temperature, salinity and light irradiance available over the ship’s network was recorded as-is by the SeaFlow computer. Any missing ship data were retrieved from the Rolling Deck to Repository.

### Data analysis

**Fig. 2:**
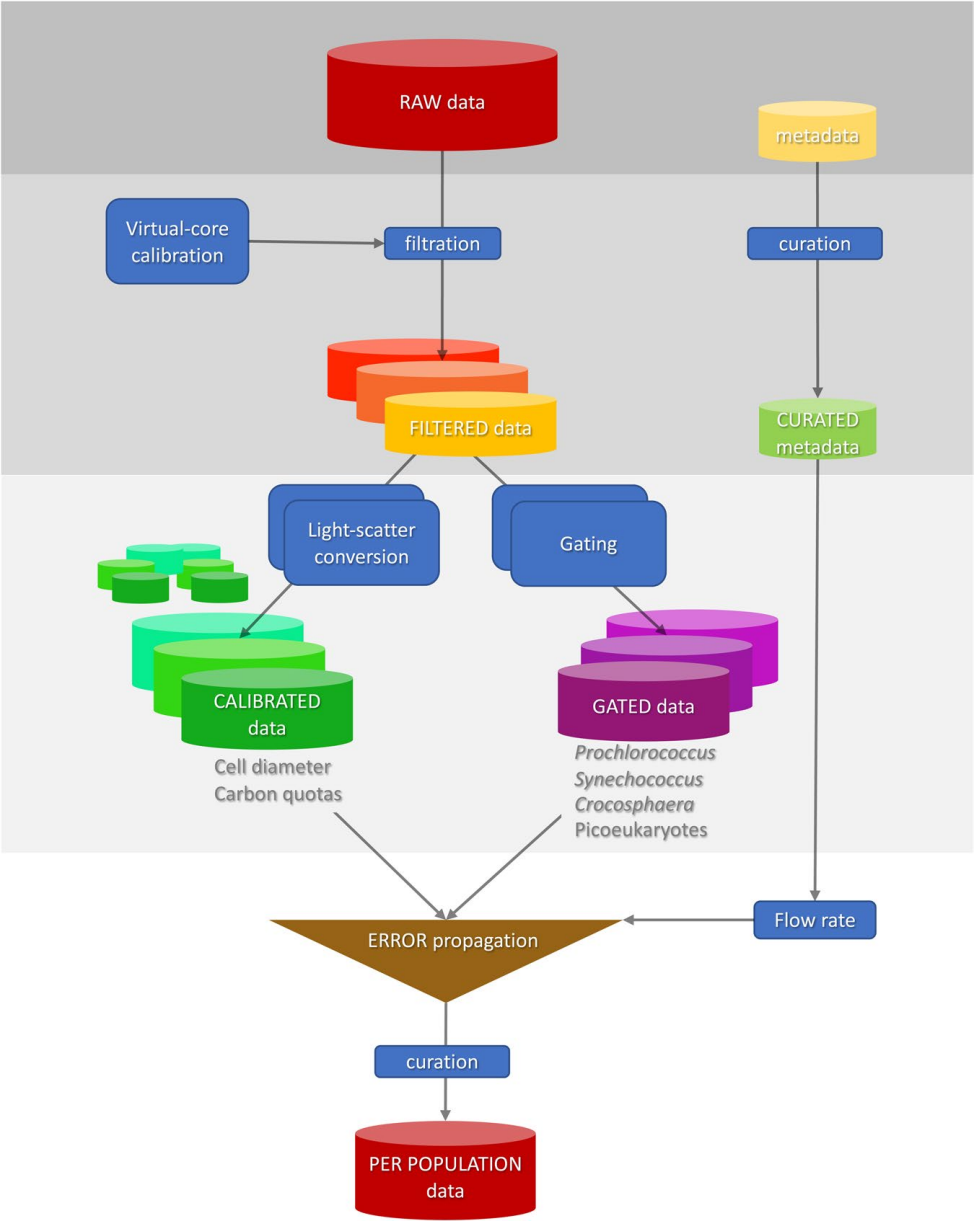
Representation of the workflow starting from the raw data source to the curated per-population SeaFlow data. Classified data is the per cell forward light scatter and fluorescence for different populations and the calibrated data is the derived per equivalent spherical and cellular carbon content.

**Fig. 3:**
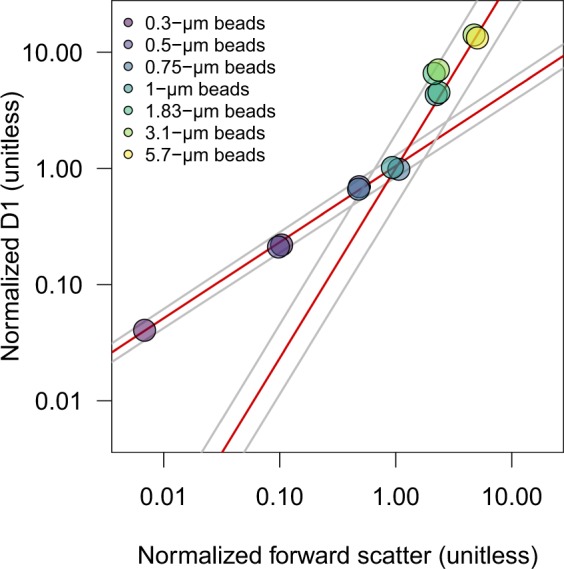
Calibration of optimally-positioned particles. Optical properties of optimally-positioned calibration beads show a linear relationship between the forward scatter and the position-sensitive detectors (D1) normalized to 1-*μ*m calibration beads, which is represented by the two linear regression models (red lines). Grey lines represent the 95% confidence interval of the two regression models.

Four data processing steps are employed to transform raw SeaFlow data into processed data (Fig. 2). First, the filtration step identifies in-focus particles positioned within the SeaFlow virtual core^8^, a cross-sectional area within the sample stream determined by the field of view of the optical system. This field of view is a function of the magnification of the objective-tube lens system and the width of the field stop. Two position detectors (D1 and D2) determine the lateral position of a particle. Particles that scatter light equally on both detectors (aligned particles) and scatter more in the forward direction than on the two position detectors (in-focus particles) are considered optimally-positioned particles (OPP). The relationship between forward scatter and the two position detectors of OPP can be described by two linear regression models intersecting at the 1-*μ*m calibration bead coordinates (Fig. 3). The uncertainties around the two slopes of the linear regression models are used to assign a confidence interval for each OPP (2.5%, 50% or 97.5% interval confidence). Each data file is linked to a unique filtration identification number that refers to the parameters used to discriminate OPP.

Second, OPP are classified into cell populations by forward scatter (457/50 bandpass filter), red fluorescence (572/28 bandpass filter) and orange fluorescence (692/40 band-pass filter). Sequential manual gating is used to cluster *Synechococcus*, small-sized *Crocosphaera* and 1-*μ*m calibration beads (Invitrogen F8823), as they each have distinguishing optical characteristics that do not overlap with other cell populations. *Prochlorococcus* particles are clustered using a supervised clustering algorithm that emulates a sequential bivariate gating strategy based on cell density^9^. High forward scatter particles with high red fluorescence were classified as “picoeukaryote” phytoplankton. Each data file is linked to a unique gating identification number that refers to the coordinates and analysis parameters used for particle classification. Cell abundance is calculated by dividing the number of particles in each population by the volume of the virtual core, which is estimated by the ratio of OPP to the total detected particles and by the volume of the sample analyzed by the instrument^8^. The sample volume is obtained after calibration of the water stream flow rate. Standard error of cell abundance represents the uncertainties in flow rate calibration.

Third, the equivalent spherical diameter (ESD) of individual cells is estimated from SeaFlow-based light scatter by the application of Mie light scatter theory to a simplified optical model. Since the optical geometry of the SeaFlow is complicated by scatter occurring within the sample stream, an optimization procedure was used to minimize differences between the measured forward scatter and the scatter intensity predicted by Mie light scatter of homogeneous spherical particles. The ESD of each phytoplankton cell was estimated from the optimized Mie model based on three refractive indices (1.35, 1.38 and 1.41) that cover the range applicable to marine phytoplankton^11^, relative to refractive index of seawater (1.34).

In the final step, carbon quotas were estimated from ESD using the equation *fgC cell*^−1^ = 0.261 × *Volume*^0.860^ ^10^, assuming spherical particles.

### Quality control procedure

The stability of stream pressure and the rate of particles detected per second are used to evaluate instrument performance. Data files are identified as outliers if the stream pressure deviates by more than 5% of the mean value for a given cruise or if data acquisition exceeds 18,000 particles per second (corresponding to 200–500 particles per second in the virtual core), when coincidence of particles is likely^8^. The quality of estimates for ESD, carbon quotas and cell abundance was assessed by applying the Chauvenet criterion^12^, which defines outliers as data points falling outside a band around the mean corresponding to a probability of 1 − 1/(2N) (where N = total number of data points).

## Data Records

The dataset is a compilation of data assembled from different research cruises conducted since 2010. Each data record represents the cell abundance, median, 25% and 75% percentile of optical properties (chlorophyll and phycoerythrin fluorescence, forward scatter), ESD and carbon quotas for each population estimated at a certain point in space and time. Each data record belongs to a cruise, with cruise identification retrieved from the Rolling Deck to Repository, and is linked to its associated metadata such as time, location, depth, sea surface temperature and salinity, and PAR. Online-only Table 1 lists the variables, their definition and units. The dataset is accessible as a.csv file through Zenodo open access research data repository^13^.

## Technical Validation

### Equivalent spherical diameter and carbon quotas

**Fig. 4:**
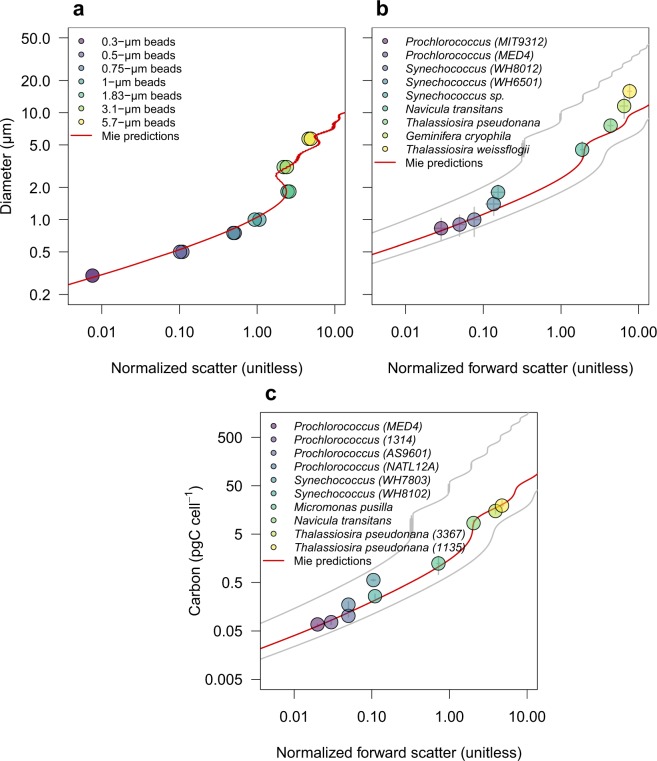
Calibration of forward scatter measurements. Relationship between forward scatter normalized to 1-*μ*m calibration beads measured by SeaFlow and (**a**) diameter of calibration beads, (**b**) equivalent spherical diameter of phytoplankton cultures and (**c**) carbon quotas estimated with independent methods. Diameters of calibration beads were provided by the manufacturer while diameters of phytoplankton type were from electronic particle counter measurements; carbon quotas was determined by bulk measurements of particulate carbon normalized by cell number. Red lines represent Mie-based predictions using a refractive index of 1.60 (**a**) or 1.38 (**b**,**c**) and 1.35 and 1.41 for grey lines, relative to the refractive index of seawater (1.34).

The optimized Mie theory was applied to SeaFlow-based scattering measurements of calibration beads of known refractive index (1.60) and diameter (0.3, 0.5, 0.75, 1, 1.83, 3.1 and 5.7 *μ*m). Mie-predicted bead diameters were in good agreement with diameters provided by the manufacturer (R^2^ = 0.98, p < 0.0001) (Fig. 4a).

To evaluate the applicability of Mie-predicted cell diameters to phytoplankton cells, a Coulter Counter Multisizer equipped with a 15-*μ*m and 30-*μ*m orifice was used to measure cell diameters of axenic, exponentially growing cyanobacteria (*Prochlorococcus* MIT9312 and MED4, *Synechococcus* WH8012, WH6501 and sp.) and eukaryotic phytoplankton (the diatoms *Navicula transitans*, *Thalassiosira pseudonana*, *Thalassiosira weissflogii and the crytophyte Geminifera cryophila*) under non-limiting light conditions (150 *μ*mol quanta m^−2^ s^−1^). These independent measurements were then compared to the equivalent spherical diameter derived from the Mie-based lookup table. The Mie-predicted ESD using the mid-range refractive index for phytoplankton (1.38) was in good agreement with observations (R^2^ = 0.96, p < 0.0001), however discrepancies were observed for the diameter of the larger phytoplankter *T. weissflogii*), suggesting a higher refractive index for this organism.

A second set of experiments was conducted to compare measurements of carbon quotas with those estimated from Mie-predicted ESD. Carbon per cell was determined for 6 axenic cyanobacteria cultures (*Prochlorococcus* MED4, MIT9312, AS9601 and NATL12A, *Synechococcus* WH7803 and WH8012) and 4 different eukaryotic phytoplankton cultures (*Micromonas pusilla*, *Navicula transitans*, *T. pseudonana* 3367 and 1135). Particulate C and N collected on pre-combusted 0.3-*μ*m GF-75 or 0.7-*μ*m GF/F filters were analyzed on a Carlo Erba CHNS analyzer (model NA1500) in the Oregon State University Stable Isotope Laboratory using cystine (29.99% C and 11.66% N by weight) as the primary standard. For each culture, aliquots of growth media filtered through three pre-combusted GF-75 and GF/F glass fiber filters were used as blanks to correct for background carbon concentration on filters before filtration and DOC adsorption onto filters. Carbon quotas were obtained by normalizing the concentrations of blank-corrected particulate carbon to cell abundance measured with a BD Influx cell sorter. Mie-predicted ESD based on light scatter measurements from SeaFlow was converted to carbon quotas using the equation *fgC cell*^−1^ = 0.261 × *Volume*^0.860^ ^11^, assuming spherical particles. We found that carbon quotas were in good agreement with our light scatter-based estimates using a refractive index for phytoplankton of 1.38 (Fig. 4c) (R^2^ = 0.96, p < 0.0001), consistent with our ESD results (Fig. 4b).

### Cell abundances

**Fig. 5:**
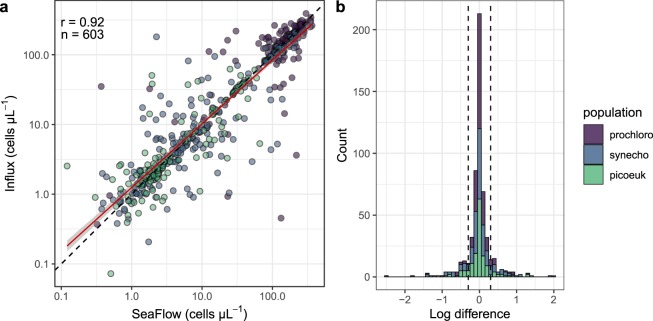
Comparison of cell counts. (**a**) Abundances of eukaryotic phytoplankton (picoeuk) *Prochlorococcus* (prochloro), *Synechococcus* (synecho) obtained with SeaFlow were compared with those obtained with a BD Influx flow cytometer. Samples analyzed with the Influx were collected from Niskin bottles and fixed with electron grade glutaraldehyde at a 0.25% final concentration while samples analyzed by the SeaFlow were collected from the ship’s underway system and were not fixed. The linear regression (red line, slope = 0.91), coefficient of correlation (R = 0.92), number of observations (n), and dashed line representing the 1:1 slope are shown. (**b**) Frequency distribution of percent discrepancy in abundance estimates between the two instruments, dashed lines representing the 25% discrepancy.

The abundance of cells within a given phytoplankton population is dependent on the ratio of OPP to the total detected particles^8^. While a single linear regression was previously used to discriminate OPP^8^, here we applied the combination of two linear regression models, which better defined the relationship between forward light scatter and the position-sensitive detectors (Fig. 3) for particles less than or greater than 1 *μ*m in ESD. We compared the resulting SeaFlow-based cell abundances of *Prochlorococcus*, *Synechococcus* and eukaryotic phytoplankton (<5 *μ*m in ESD) with fixed samples collected concurrently on 17 cruises (n = 201) and measured on a BD Influx Cytometer. Particle counts for the three phytoplankton groups were in good agreement between the two instruments (R = 0.92, n = 603, slope of the regression line = 0.91) (Fig. 5), with 74% of the estimates (444/603) showing less than a 2-fold difference. 3% (17/603) of the estimates showed 1–2 order of magnitude difference, likely reflecting natural variability rather than instrument counting error.

## Data Availability

Raw SeaFlow data are analyzed using our custom R package available on Github at https://github.com/armbrustlab/popcycle. The repository also includes a tutorial on the use of the software. Additional Github repositories are available for the virtual-core calibration, conversion of light scattering to cell size and conversion of light scattering to carbon quotas.

## Online-only Table

**Online-only Table 1:** List of variables, variable definition and unit used in the SeaFlow data.

Variable	Variable definition	Units
*cruise*	cruise identification	
*time*	time of sample collection	%Y-%m-%dT%H:%M:%S UTC
*lat*	latitude	decimal degree North
*lon*	longitude	decimal degree East
*temp*	uncalibrated sea surface temperature	deg C
*sal*	uncalibrated sea surface salinity	psu
*par*	uncalibrated surface Photosynthetic active radiation	µmol quanta m^−2^ s^−1^
*quantile*	OPP confidence interval	2.5 = conservative approach
		50 = standard approach
		97.5 = permissive approach
*pop*	population	prochloro (*Prochlorococcus*)
		synecho (*Synechococcus*)
		croco (*Crocospheara*)
		picoeuk (phytoplankton <5 µm ESD)
		beads (1 µm calibration beads, Invitrogen F8823)
		unknown (unclassified particles)
*chl_1q*	1^st^ quartile of chlorophyll fluorescence	unitless
*chl_med*	median of chlorophyll fluorescence	unitless
*chl_3q*	3^rd^ quartile of chlorophyll fluorescence	unitless
*pe_1q*	1^st^ quartile of phycoerythrin fluorescence	unitless
*pe_med*	median of phycoerythrin fluorescence	unitless
*pe_3q*	3^rd^ quartile of phycoerythrin fluorescence	unitless
*fsc_1q*	1^st^ quartile of forward scatter	unitless
*fsc_med*	median of forward scatter	unitless
*fsc_3q*	3^rd^ quartile of forward scatter	unitless
*diam_lwr_1q*	1^st^ quartile of ESD using low refractive index	µm
*diam_lwr_med*	median of ESD using low refractive index	µm
*diam_lwr_3q*	3^rd^ quartile of ESD using low refractive index	µm
*diam_mid_1q*	1^st^ quartile of ESD using mid refractive index	µm
*diam_mid_med*	median of ESD using mid refractive index	µm
*diam_mid_3q*	3^rd^ quartile of ESD using mid refractive index	µm
*diam_upr_1q*	1^st^ quartile of ESD using high refractive index	µm
*diam_upr_med*	median of ESD using high refractive index	µm
*diam_upr_3q*	3^rd^ quartile of ESD using high refractive index	µm
*Qc_lwr_1q*	1^st^ quartile of carbon quotas using low refractive index	pg cell^−1^
*Qc_lwr_med*	median of carbon quotas using low refractive index	pg cell^−1^
*Qc_lwr_mean*	mean of carbon quotas using low refractive index	pg cell^−1^
*Qc_lwr_3q*	3^rd^ quartile of carbon quotas using low refractive index	pg cell^−1^
*Qc_mid_1q*	1^st^ quartile of carbon quotas using mid refractive index	pg cell^−1^
*Qc_mid_med*	median of carbon quotas using mid refractive index	pg cell^−1^
*Qc_mid_mean*	mean of carbon quotas using mid refractive index	pg cell^−1^
*Qc_mid_3q*	3^rd^ quartile of carbon quotas using mid refractive index	pg cell^−1^
*Qc_upr_1q*	1^st^ quartile of carbon quotas using high refractive index	pg cell^−1^
*Qc_upr_med*	median of carbon quotas using high refractive index	pg cell^−1^
*Qc_upr_mean*	mean of carbon quotas using high refractive index	pg cell^−1^
*Qc_upr_3q*	3^rd^ quartile of carbon quotas using high refractive index	pg cell^−1^
*abundance*	cell abundance	cells µL^−1^
*abundance_se*	standard error of cell abundance	cells µL^−1^
*flag*	outliers	0 = Quality data
		1 = Instrument issue
		2 = OPP filtration issue
		3 = Gating issue
